# Large surface deformation due to thermo-mechanical effects during cryopreservation by vitrification – mathematical model and experimental validation

**DOI:** 10.1371/journal.pone.0282613

**Published:** 2023-03-09

**Authors:** Devarsh M. Vispute, Prem K. Solanki, Yoed Rabin

**Affiliations:** Biothermal Technology Laboratory, Department of Mechanical Engineering, Carnegie Mellon University, Pittsburgh, Pennsylvania, United States of America; National Textile University, PAKISTAN

## Abstract

This study presents a simplified thermal-fluids (TF) mathematical model to analyze large surface deformations in cryoprotective agents (CPA) during cryopreservation by vitrification. The CPA deforms during vitrification due to material flow caused by the combined effects of thermal gradients within the domain, thermal contraction due to temperature, and exponential increase in the viscosity of the CPA as it is cooled towards glass transition. While it is well understood that vitrification is associated with thermo-mechanical stress, which might lead to structural damage, those large deformations can lead to stress concentration, further intensifying the probability to structural failure. The results of the TF model are experimentally validated by means of cryomacroscopy on a cuvette containing 7.05M dimethyl sulfoxide (DMSO) as a representative CPA. The TF model presented in this study is a simplified version of a previously presented thermo-mechanics (TM) model, where the TM model is set to solve the coupled heat transfer, fluid mechanics and solid mechanics problems, while the TF model omits further deformations in the solid state. It is demonstrated in this study that the TF model alone is sufficient to capture large-body deformations during vitrification. However, the TF model alone cannot be used to estimate mechanical stresses, which become significant only when the deformation rates become so small that the deformed body practically behaves as an amorphous solid. This study demonstrates the high sensitivity of deformation predictions to variation in material properties, chief among which are the variations of density and viscosity with temperature. Finally, this study includes a discussion on the possibility of turning on and off the TF and TM models in respective parts of the domain, in order to solve the multiphysics problem in a computationally cost-effective manner.

## Introduction

Despite remarkable advancements in organ transplantation, the shortage of suitable organs on demand is the major obstacle, preventing this life-saving treatment from reaching its potential. Roughly 36,000 organ transplants were performed in US in 2018, whereas the total number of patients on the waitlist for transplant was more than 110,000 [1]. The preservation of tissues and organs can provide the ability to replace organs and tissues on demand, saving millions of lives each year, which is a public health benefit on par with curing cancer [2]. The preservation of organs and tissues has thus been described as “the supply line for organ transplantation” [3].

While cryopreservation has successfully been implemented for preservation of small-sized specimens (in the scale of μm to mm), preservation of large-sized tissues and organs (in a scale of cm and above) requires overcoming challenges related to toxicity of cryoprotective agents (CPA) [4], ice formation [5], chilling and ischemic injuries [6], and thermomechanical stress [7–11]. Cryopreservation of tissues and organs by means of vitrification (*vitreous* in Latin means *glassy*) is a promising ice-free preservation alternative for large size biological specimens [12, 13].

While suppression of ice crystallization is key for successful vitrification [5], it also depends on a delicate balance between the competing needs to reduce the toxicity effects of the CPA [4], and to keep thermo-mechanical stresses in safe levels [7, 14–16]. Thermo-mechanical stress (or thermal stress for simplicity) is driven by the natural tendency of all materials to change volume with temperature, with the thermal expansion coefficient as its measure. Thermal stress develops when the thermal expansion is constraint, and structural damage will follow when the stress reaches excessive levels [7, 14, 15, 17–19]. Thermal stress can also arise due to thermal expansion associated with phase transition [20] (partial crystallization), differential thermal expansion within the material due to temperature gradients [8], and thermal expansion mismatch between the specimen and the container [21].

Thermal expansion may also drive fluid flow during vitrification, causing the specimen to deform at a rate which is affected by the viscosity of the material [17]. The viscosity of the CPA increases exponentially by fifteen orders of magnitude during the vitrification process [22], from a water-like viscosity level at room temperature, to such a high value around the glass transition temperature, that flow is practically arrested and the material behaves like an amorphous solid. Due to the combined effect of temperature gradients during the inward cooling process of the specimen, and variations in thermal expansion and viscosity with temperature, dramatic deformation at free surfaces may be observed [11, 23–25]. However, since these surface deformations dissipate during rewarming, as the CPA regains fluid-like viscosity at higher temperatures, and since the specimens are most frequently evaluated only after recovery from cryogenic storage, such large surface deformations has eluded cryobiologists for decades.

A Proprietary *in-situ* imaging device, known as the *cryomacroscope*, was used to first observe the formation of large deformations in the shape of a surface cavity in vials and cuvettes filled with CPA [23–25]. Over the past two decades, five different types of the cryomacroscope have been developed, to facilitate visualization of physical effects like crystallization, contamination, surface deformation, photoelasticity effects, and fracturing in specimens undergoing cryopreservation protocols [23–26].

Such large deformation can increase the average stress level within the specimen and cause stress concentrations along its surfaces [11, 23–26]. The stress developed during cooling might intensify when the temperature distribution across the specimen equilibrates during cryogenic storage (i.e., residual stress), and might even further intensify at the onset of rewarming [11, 27, 28]. Previous thermomechanical stress analysis relied on extracting the deformed surface geometry from cryomacroscopy images [26]. This approach is based on the notion that large deformations occur in the CPA only when its viscosity is low and consequently stress development is negligible, while significant stresses develop only in lower temperatures, where the viscosity is high and deformations are small.

A thermo-mechanics (TM) mathematical model has been recently presented to investigate the effects of free surface deformation during vitrification [11]. The TM model is formulated to solve the related coupled problem of heat transfer, fluid mechanics, and solid mechanics. Following the good qualitative agreement of the TM model with experimental data obtained using the scanning cryomacroscopy [23, 25, 26], the current study aims at quantitative validation of the proposed model, while putting into test its underlying assumption and its sensitivity on material properties. The previous study included solid mechanics aspects to study effects such as stress concentration and the likelihood to structural damage, whereas the current study focuses on the ability to model surface deformations. With this objective in mind, the current study proposes a simplified thermal-fluids (TF) mathematical model and explores if is sufficient to capture body deformation by means of validation against experimental data. Unlike the TM model, the TF model is formulated to solve the coupled problem of heat transfer and fluid mechanics only, excluding solid-mechanics effects, and thereby reducing computation complexity and accelerating computation runtime. In summary, the unique contribution of the current study is twofold: (i) presenting a simplified mathematical model for analysis of free surface deformation during vitrification, and (ii) validating the model against experimental results obtained on the cryomacroscope platform.

## Methods and materials

### Experimental apparatus

**Fig 1:**
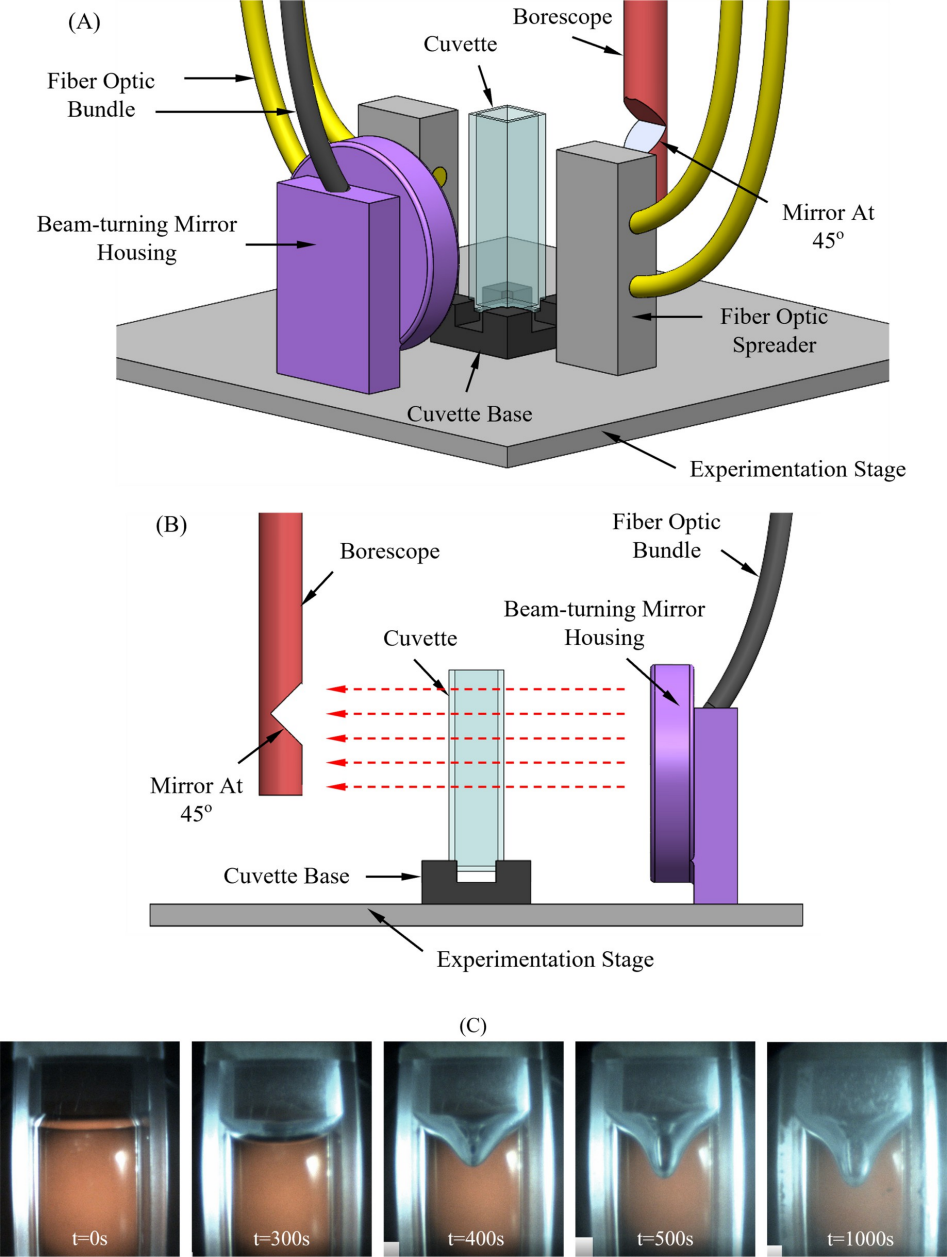
(A) Schematic illustration of the cryomacroscope. (B) Side view illustration showing the path of light passing through the cuvette using red arrows. (C) Selected images displaying surface deformation at various times during the vitrification process subject to a constant cooling rate of 25°C/min from an initial temperature of 10°C down to final temperature of -125°C. The cuvette has outer dimensions of 12.5 mm × 12.5 mm × 45 mm, filled with 2.8 ml of 7.05M DMSO.

The scanning cryomacroscope was used in this study, which is a device tailored for *in situ* visualization of macro-scale physical events, such as ice crystallization, fracturing, thermal strain (photoelasticity), and contamination that may occur during cryopreservation processes [23–26]. Since this device has been presented previously in various configurations [23, 25, 26], the specific setup is described here in brief only, for completeness of the presentation. The cryomacroscope is designed to retrofit as the lid of a commercially available controlled-rate cooler (Kryo 10, Planar PLC, UK). Due to the harsh environment in the cooling chamber, visualization is achieved by means of a borescope, where all electronic components, including the camera, its scanning mechanism, and the light sources are positioned external to the cooling chamber. Fig 1(A) displays the experimentation stage of the cryomacroscope, where the imaging target is a CPA-filled cuvette (a rectangular vial with superior optical properties) [23]. Scanning of the cuvette may be assisted by direct or background illumination, facilitated by fiber-optics bundles extending up to the specimen stage from the light sources placed outside the cooling environment.

The red arrows in Fig 1(B) display the direction of view, where the image is reflected through a 45° mirror to a CCD camera [23, 26]. A stepper motor and a mount-and-carriage system for the camera setup allows for vertical scanning of the entire cuvette. The thermal history of the cooling chamber is obtained with a K-type thermocouple placed in the freestream flow of coolant surrounding the cuvette [23–25]. Control of the various components of the cryomacroscope, as well as monitoring of images and temperatures in real-time, are carried out using a proprietary cryomacroscope control code (C^3^) [23–26]. The same software is also used for post processing to create a digital video overlaying time and temperature data. Fig 1(C) displays snapshots of the surface deformation at selected points in time during cooling.

### Benchmark data

Reference datasets for surface deformations were obtained while experimenting on dimethyl sulfoxide (DMSO) as a vitrifying material, which is the key ingredient in many CPA cocktails [29]. Specifically, 7.05 M DMSO solution has been demonstrated previously as a reference solution to study thermo-mechanics effects in high concentration CPAs, such as VS55 and DP6, which drew significant attention over the years [25]. Consistent with previous studies [23, 25], polystyrene cuvettes (Plastibrand, BRAND Gmbh + Co. KG, Germany) are used for the experiments, having outer dimensions of 12.5 mm × 12.5 mm × 45 mm and wall thickness of 1.25 mm, Fig 1. Relevant physical properties for the 7.05 M DMSO and polystyrene are listed in Table 1.

**Table 1:** Material properties of 7.05M DMSO and polystyrene used in this study (temperatures are in °C).

Property	Material	Value/Function	Ref.
Density, kg/m^3^	CPA	1090–0.6922T+0.000257T^2^	[29]
	Cuvette	1055–0.26T	[30]
Thermal conductivity, W/m-°C	CPA	0.356+7.42×10^-4^T-1.29×10^-6^T^2^-6.87×10^-8^T^3^-2.95×10^-10^T^4^	[31]
	Cuvette	0.14+1.3×10^-4^T	[32]
Thermal Expansion Coefficient, 1/°C	CPA	1.1×10^−5^	[33]
	Cuvette	8×10^−5^	[30]
Specific Heat, J/kg-°C	CPA	2804+4.205T−0.054T^2^−4.902×10^−5^T^3^	[24]
	Cuvette	1121+3.94T	[34]
Heat Transfer Coefficient, W/m^2^-°C	Free, *h*_*1*_	10	[24]
	Forced, *h*_*2*_	350	

**Fig 2:**
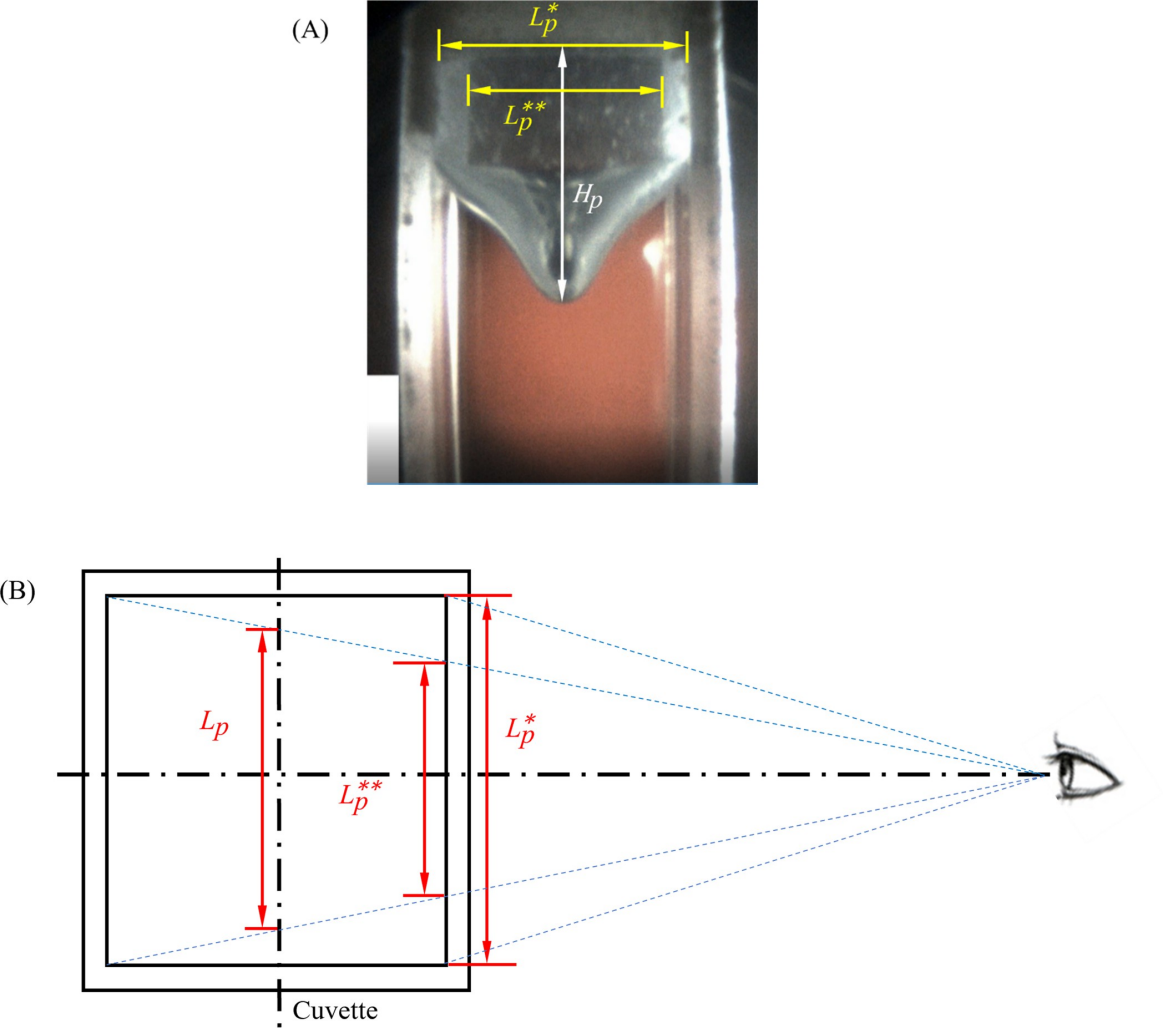
Parameters used for the analysis of experimental measurements, Eqs (1) and (2). (A) front view of the cuvette from the cryomacroscope, and (B) top view of the cuvette illustrating the scaling effect (not drawn to scale). The calculated inner width of the cuvette on the front face, Lp*, is 10.18 ± 0.11 mm for image in (A), while the experimentally measured width is 10 ± 0.1 mm.

Two types of datasets were collected for the comparison of computer modeling results with experimental data: (i) the shape of the deformed surface across the center plane of the cuvette; and (ii) the transient displacement of the CPA surface along the centerline of the cuvette, that is the point of maximum deformation. With reference to Fig 2(A), the free surface displacement along the centerline of the cuvette is calculated by:

$$u_{s}(t)=H_{e}(t)-H_{e}(0);\;H_{e}=\frac{H_{p}L_{e}}{L_{p}}$$ (1)

where *H* is height from a refence line marked on the cuvette, *L* is the inner width of the cuvette, *t* is time, and the indices *e* and *p* refer to experimental value and its appearance on the computer screen, respectively.

A special attention is paid to the calculation of *L*_*p*_ since there is no reference width along the center plane of the cuvette. Here, due to the symmetry of the problem, and since given the change in the apparent width with the depth of field, the reference width is estimated as:

$$L_{p}=\frac{1}{2}(L_{p}^{*}+L_{p}^{**})$$ (2)

where *L*_p_* and *L*_p_** are the apparent inner widths of the cuvette on the front and back surfaces of the cuvette, Fig 2(B).

The two most significant sources of uncertainty in displacement measurements, using the scheme described above are: (i) uncertainty in distance measurements on the computer screen, which accounts for screen resolution, the computation measurement tool, and human error; and (ii) reference measurement of the actual cuvette width with a caliper, which is assumed to be the same as the uncertainty in the specific caliper measurements, *δL*_*e*_ = ±0.1 mm.

The uncertainty in measurements from the computer screen, *δH*_*p*_, is estimated as twice the standard deviation in measuring a cavity depth, *H*_*p*_, in a series of independent measurements:

$$\delta H_{p}=2\sqrt{\frac{\sum_{i=1}^{n}{\left(H_{p,i}-\bar{H_{p}}\right)}^{2}}{n-1}}$$ (3)

where *δ* refers to uncertainty, *n* is the number of repetitions, *i* is an index, and the bar represents an average value. Based on 10 repetitions, the uncertainty in depth measurements is estimated as ±0.11 mm or 0.94% of a cavity depth of 12.1 mm from the reference line, corresponding to the maximum deformation for the cooling rate of 20°C/min at the temperature of -125°C.

The uncertainty in width measurements is calculated as:

$$\delta L_{p}=\sqrt{3{\left(\delta H_{p}\right)}^{2}}$$ (4)

where the uncertainties in vertical and horizontal measurements on the computer screen are similar, while the factor 3 corresponds to evaluating *L*_*p*_ as an average of two additional measurements, Eq (2). The uncertainty in width measurements, *δL*_*p*_ is estimated as ±0.19 mm, corresponding to the maximum deformation for the cooling rate of 20°C/min at the temperature of -125°C.

Finally, the resulting uncertainty in the cavity depth measurements can be calculated as:

$$\delta u_{s}=\sqrt{{\left(\frac{\partial u_{s}}{\partial H_{p}}\delta H_{p}\right)}^{2}+{\left(\frac{\partial u_{s}}{\partial L_{p}}\delta L_{p}\right)}^{2}+{\left(\frac{\partial u_{s}}{\partial L_{e}}\delta L_{e}\right)}^{2}}$$ (5)

where the error bars displayed with the experimental data in this study equal ± 2*δu*_*s*_.

While the estimated uncertainty in Eq (5) is associated with the analysis of a single experiment, the repeatability in experimentation should also be considered when comparing experimental observation with mathematical modeling. For this purpose, four experiments were performed under the same cooling rate and final temperature of 20°C/min and -125°C, respectively. Using Eq (1), the maximum depth cavity for this set of experiments was found to be 5.48 ± 0.15 mm, or variability among experiments of 2.74% of full depth.

### Mathematical modeling

#### Heat transfer model

The heat transfer model accounts for combined heat conduction and free convection due to the temperature-dependent density, while ignoring the viscous heat dissipation [35]:

$$\rho c_{p}\left(\frac{\partial T}{\partial t}+v.\nabla T\right)=\nabla \cdot (k\nabla T)$$ (6)

where *ρ* is the density, *c*_*p*_ is the specific heat, *t* is the time, *T* is the temperature, ***v*** is the velocity field, *k* is the thermal conductivity, and bold symbols signify vectorial quantities. Heat transfer by forced convection is assumed on the outer surface of the cuvette [35]:

$$-\hat{n}\cdot \left(k\nabla T\right)=h(T_{c}-T_{\infty })$$ (7)

where

$\hat{n}$ is a unit normal to the outer surface, and the subscripts *c* and *∞* denote the outer wall surface of the cuvette and cooling chamber environment, respectively. The overall heat transfer coefficient between the cuvette wall and the cooling chamber environment, *h*, was measured experimentally in a previous study [24]. Continuity in temperature and heat flux is assumed on all interfaces between the container wall and CPA [11, 35].

#### Fluid mechanics model

The Navier-Stokes equation is used to model CPA flow, while assuming that the inertial forces are negligible in comparison with the viscous forces (i.e., creeping flow) [36]:

$$\rho \frac{\partial v}{\partial t}=\nabla \cdot \left[-pI+\mu \left(\nabla v+{\left(\nabla v\right)}^{\prime }\right)-\frac{2}{3}\mu \left(\nabla \cdot v\right)I\right]+\rho g$$ (8)

where *p* is the pressure, ***I*** is the identity matrix, *μ* is the dynamic viscosity, ***g*** is the gravitational acceleration, the prime denotes matrix transposition. The conservation of mass is given by [36]:

$$\frac{\partial \rho }{\partial t}+\nabla \cdot \left(\rho v\right)=0$$ (9)

The coupling between the heat transfer and fluid mechanics models comes about in two ways: (i) by implementing temperature-dependent viscosity and density in Eqs (8)–(9), and (ii) by using the solution to the velocity field in heat transfer calculations based on Eq (6).

**Fig 3:**
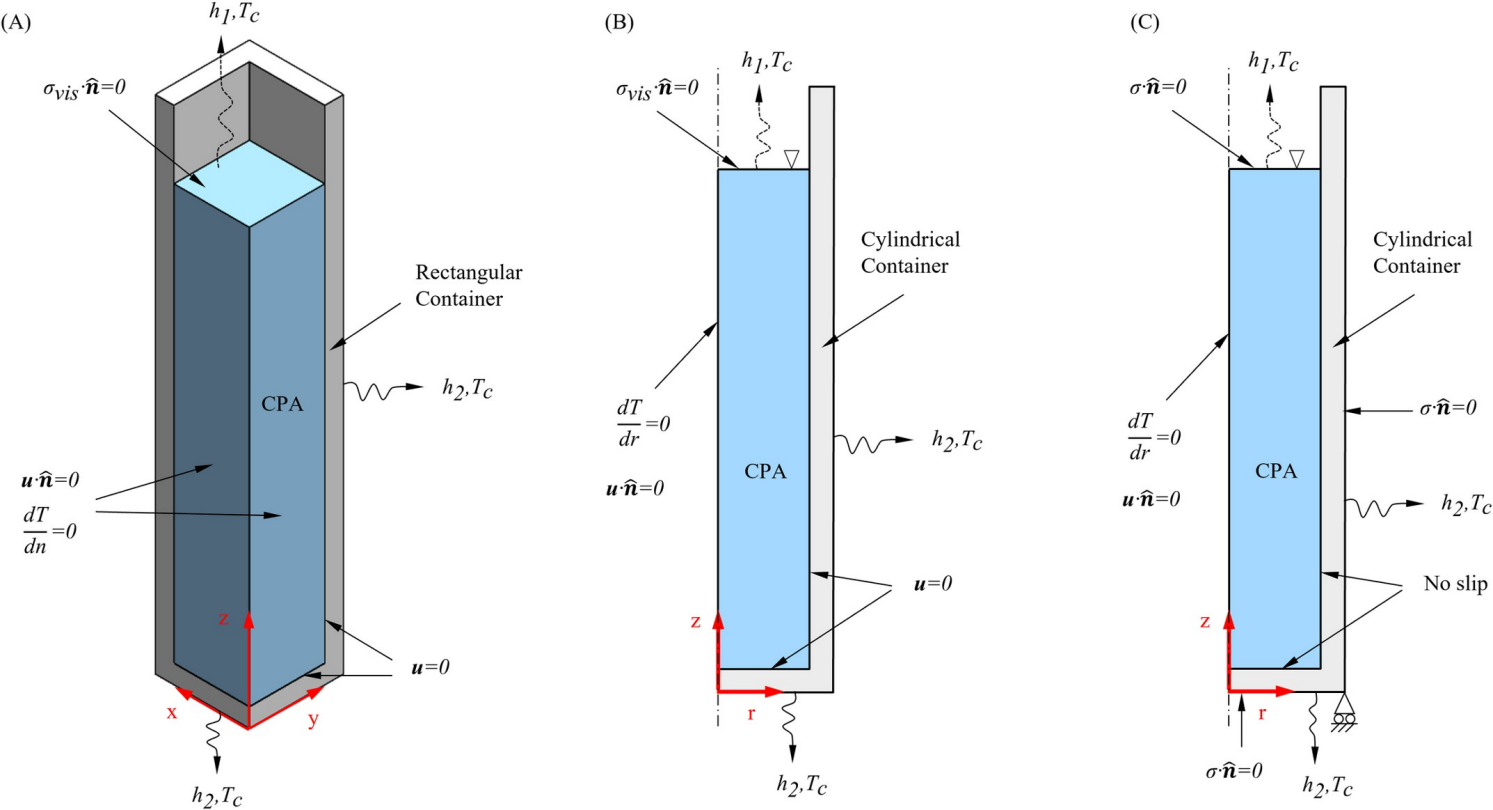
Schematic illustration of the geometric models and applied boundary conditions for FEA. (A) 3D geometric model and boundary conditions for the TF model; (B) 2D axisymmetric geometric model and boundary conditions for the TF model; (C) 2D axisymmetric geometric model and boundary conditions for the TM model; where *σ*_*vis*_ denotes stress due to viscosity effects in the TF model.

Special attention is paid to the free surface boundary condition, Fig 3(B). The normal stress at the free surface (i.e., at the CPA-air interface) is assumed zero, while surface tension is neglected [36]:

$$|\left[-pI+\mu \left(\nabla v+{\left(\nabla v\right)}^{T}\right)-\frac{2}{3}\mu \left(\nabla \cdot v\right)I\right]\cdot \hat{n}=0$$ (10)

Lastly, a no slip boundary condition is assumed on all solid surfaces:

$$v_{w}=0$$ (11)

#### Solid mechanics model

The vitrifying CPA is modeled as a Maxwell fluid [10, 11, 24, 26, 27, 37]:

$$\dot{\varepsilon }={\dot{\varepsilon }}_{elastic}+{\dot{\varepsilon }}_{creep}+{\dot{\varepsilon }}_{thermal}$$ (12)

where the elastic strain, *ε*_*elastic*_, the creep strain, *ε*_*creep*_, and the thermal strain, *ε*_*thermal*_, are calculated by:

$${\dot{\varepsilon }}_{elastic}=\frac{1}{E}\left[\left(1+v\right)\dot{\sigma }-vI\cdot tr\left(\dot{\sigma }\right)\right]$$ (13)

$${\dot{\varepsilon }}_{creep}=\frac{S}{2\mu }$$ (14)

$${\dot{\varepsilon }}_{thermal}=\alpha \dot{T}I$$ (15)

and where *E* is Young’s Modulus, *ν* is Poisson’s ratio, ***σ*** is the stress, *tr* is the trace of the stress tensor, ***S*** is the deviatoric stress tensor, and *α* is the thermal expansion coefficient. Finally, consistent with current and prior experimental observations [7, 11, 24, 28], it is assumed that the CPA adheres to the container walls as it vitrifies.

### Thermal protocol

This study focuses on the cooling part of the cryogenic protocol, where two group of experiments were conducted. The first consists of a constant cooling rate from an initial temperature of 10°C down to a final temperature of -125°C, which is 7°C above the glass transition temperature of 7.05M DMSO (-132°C) [22, 33]. The variable parameter in this experimental group is the cooling rate, ranging between 10°C/min and 25°C/min, which are all above the critical cooling rate (CCR) required to avoid crystallization in 7.05M DMSO (<5°C/min) [38]. The same cooling rate of 20°C/min was kept constant in the second group, while the final temperature varied in the range of -115°C and -135°C. Either way, the specific experimental thermal history was used as an input for the respective computer modeling for benchmarking purposes.

### Computer modeling

Computer modeling was performed using the finite element analysis (FEA) commercial software package COMSOL Multiphysics (v5.6). Due to the high computation cost of such analysis, given the symmetry of the problem, and as reference for future studies, Fig 3 displays two closely related thermo-fluids problems: (A) a rectangular geometry, depicting the 3D nature of the problem, having the same dimensions as the cuvette used for experimentation (Fig 1); and (B-C) a cylinder, representing a 2D and axisymmetric problem, having the same outer diameter as the external width of the cuvette, and the same wall thickness and height. The CPA was filled up to a height of 27.5 mm in all cases. Based on solution convergence studies, the 3D geometry was discretized into 10^5^ linear elements, combining tetrahedral, prismatic, and quadrilateral, shapes based on a volume criterion and an inbuilt mesh generator in COMSOL Multiphysics. Similarly, the 2D geometry was discretized into 4×10^4^ linear triangular elements.

Modeling of the large surface deformation observed during experimentation requires a special attention, to maintain the integrity of numerical solution [39]. While a simple solution to the problem may come about by incrementally remeshing that deforming object with a mesh suitable for small deformations, the current study uses the so-called Arbitrary Lagrangian-Eulerian (ALE) approach, which permits the mesh itself to experience large deformations without compromise the accuracy of the solution, and thereby accelerates the solution runtime [11, 39]. Implementation of this solution approach requires the specification of the mesh deformation velocity on all boundaries, which requires a special attention at the free surface (the deformation along the walls is restricted). In practice, the mesh deformation velocity normal to the air-CPA interface is calculated as the normal component of the fluid velocity field from Eq (8). The mathematical model utilizes automatic time stepping with a maximum time step size of 20s, based on a convergence study.

## Results and discussion

### Experimental validation of the TF modeling

**Fig 4:**
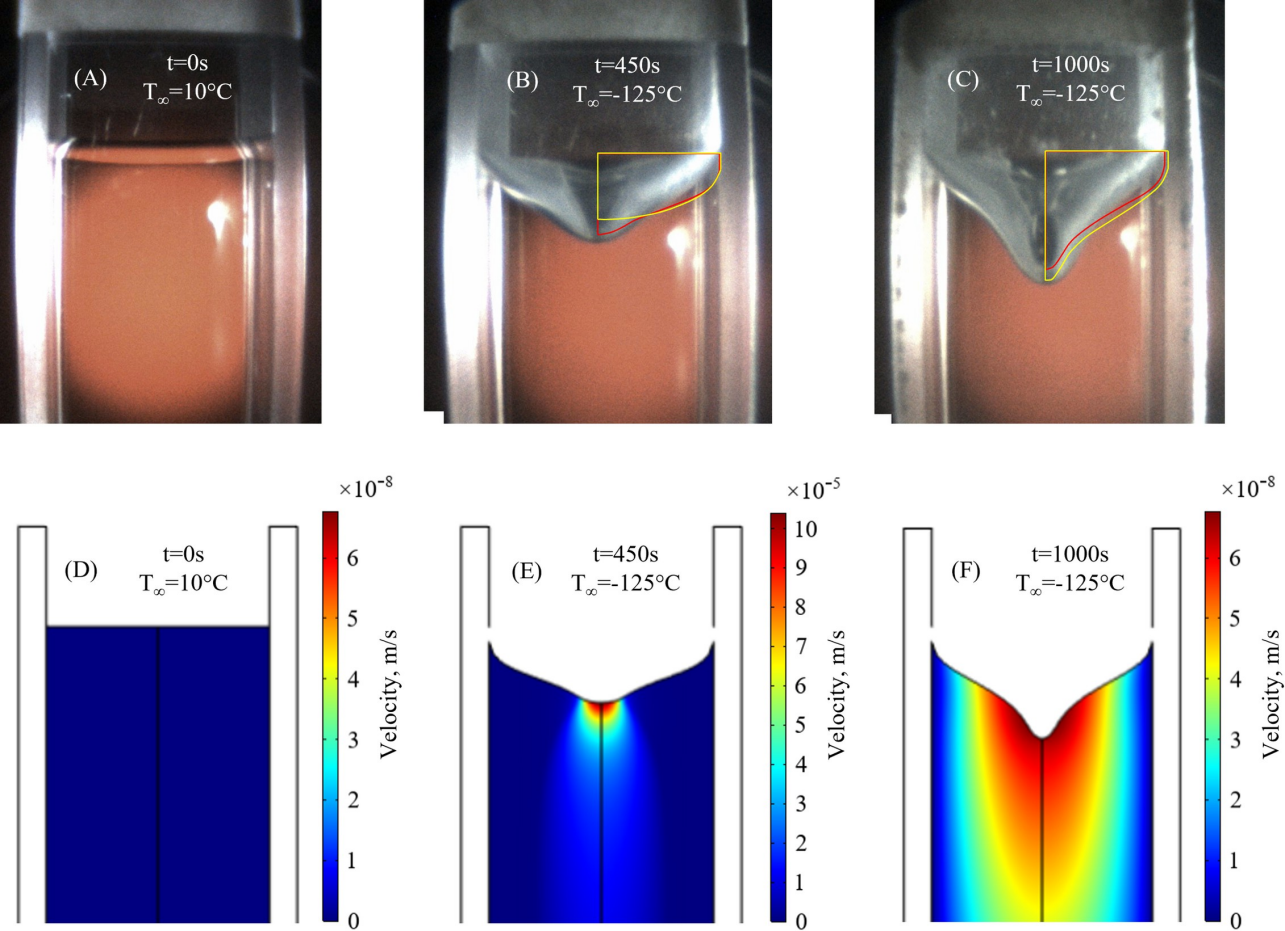
Comparison of experimental results with mathematical modeling, subject to an initial temperature of 10°C and a cooling rate 20°C/min. (A)-(C) display the snapshots of the deformed surface at different times, while (D)-(F) display the 2D TF modeling results, respectively. In addition, the overlayed red and yellow curves on the snapshots display the deformed surface base on the 2D and the 3D TF problem, respectively. Note the different velocity field scales.

**Fig 5:**
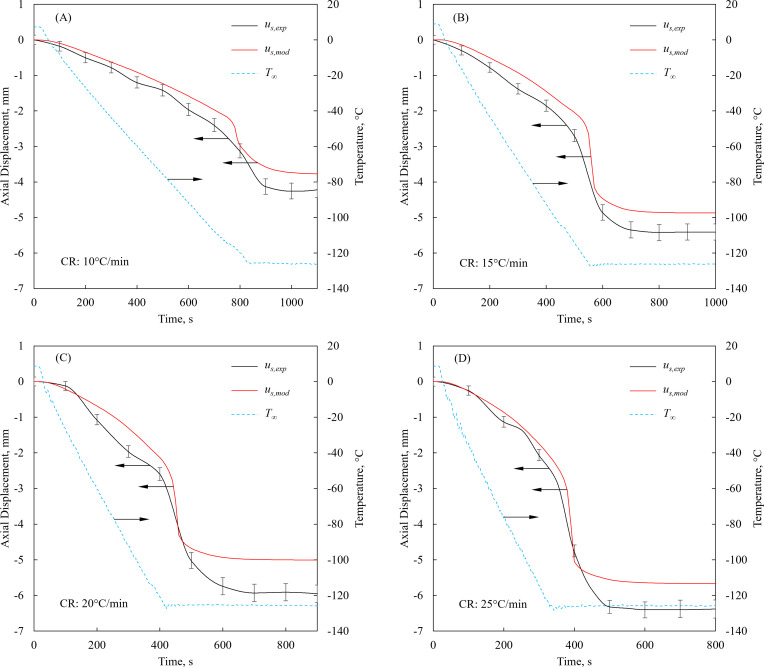
Displacement history along the centerline of the cuvette subject to a constant cooling rate (CR). The CR ranges from 10°C/min to 25°C/min from an initial temperature of 10°C, where computer modeling results are obtained for a 2D TF problem, and the subscripts *exp* and *mod* refer to experimental data and modeling results, respectively.

Fig 4 displays comparison of experimental data with 2D and 3D TF modeling results for a representative case of 20°C/min cooling rate and final temperature of -125°C. While qualitatively both the 2D and 3D cases display a similar trend of deformation, the 2D solution better captures the free surface shape earlier in the process, Fig 4(B), while the 3D solution better captures the shape at a more advanced stage, Fig 4(C). Fig 5(C) displays the free surface displacement history comparison along the centerline of the cuvette, for the same experiment, displayed in Fig 4. Furthermore, Fig 5 displays the sensitivity of the free surface deformation history to the cooling rate. The maximum displacement observed during experimentation is 4.26±0.22 mm, 5.41±0.23 mm, 6.12±0.25 mm, and 6.37±0.25 mm for cooling rates of 10°C/min, 15°C/min, 20°C/min, and 25°C/min, respectively. The maximum displacement calculated based on the 2D TF model for the same cooling rates is, 3.77 mm, 4.87 mm, 5.01 mm, and 5.67 mm, respectively. Consistent with previous observations [11, 24], the final maximum axial displacement increases with cooling rate, as can be observed from Fig 5. Faster cooling rates result in higher thermal gradients in the CPA domain [24], which explain the increase in *u*_*s*_ with increasing cooling rates [23].

From a related case study for the final temperatures of -115°C, -125°C, and -135°C, while maintaining an initial temperature of 10°C and a cooling rate of 20°C/min, the maximum final *u*_*s*_ was experimentally measured as 3.92±0.20 mm, 6.12±0.25 mm and 8.61±0.29 mm, respectively. The corresponding values based on 2D TF modeling was calculated as 3.41 mm, 5.01 mm, and 10.04 mm, respectively. It follows that the magnitude of surface deformation increases with the cooling rate and the decreasing final temperature.

The average difference between the modeling results and the experimental data in these study cases is 13.4%, while the maximum difference of 18.1% is observed for the cooling rate of 20°C/min and final temperature of -125°C. The mismatch between experimental data and simulation results can be attributed to the following sources: (i) the fish-eye effect [40] associated with the borescope optics, which can cause the center of the image to appear bulged; (ii) the surface tension effect on the initial surface shape, which deviates from the ideal flat surface modeled; (iii) uncertainty in computer modeling due to reported uncertainty in thermophysical properties [41], which is not accounted for in this study; and (iv) gaps in knowledge about material properties.

**Fig 6:**
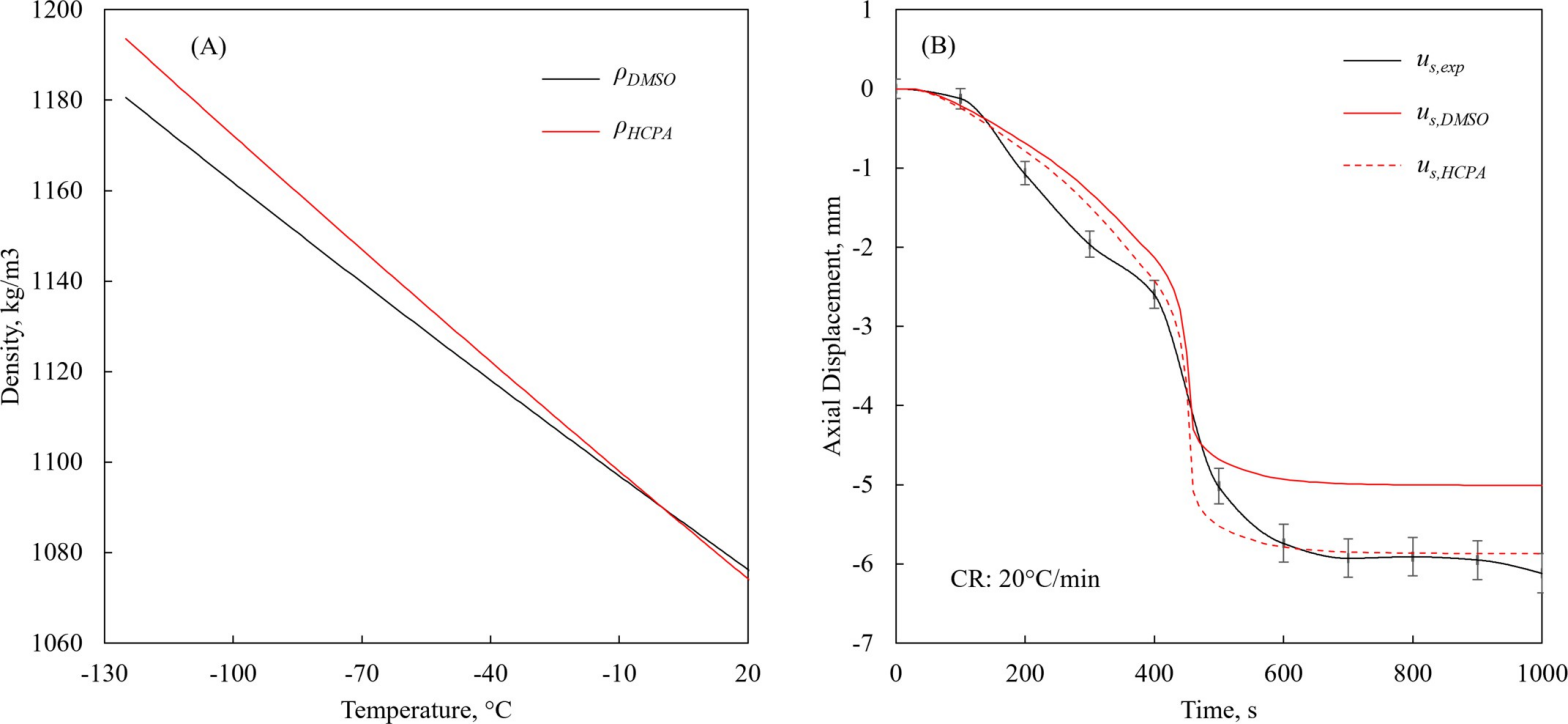
A 2D TF modeling case study on the effect of density variation with temperature, where HCPA is a hypothetical CPA having similar properties to 7.05M DMSO but 15% steeper variation. (A) temperature-dependent density; (B) experimental results used as the base for this case study, representing the worst match between modeling and experimental data in Fig 5 (cooling rate of 20F0B0C/min). The steeper-slope density curve results in absolute difference in density in the range of 0 to 0.88%, and a relative density variation of 0 to 14.5% between the temperatures 0°C to -100°C, respectively.

To study the sensitivity of the mathematical solution to variation in CPA density, a hypothetical CPA material was assumed (HCPA), which has identical thermophysical properties to 7.05M DMSO (Table 1), except for the density. The increasing density of the HCPA with the decreasing temperature is 15% steeper than that of 7.05M DMSO, as illustrated in Fig 6(A). This steeper slope results in absolute difference in density in the range of 0 to 0.88%, and a relative density variation of 0 to 14.5% between the temperatures 0°C to -100°C, respectively. Fig 6(B) displays the resulting axial displacement at the center of the cuvette, for the 2D TF model subject to initial condition of 10°C and cooling rate of 20°C/min, a case for which the experimental and modeling results show the highest difference at final temperature. Clearly, while 0.88% of measured density values is expected to be much smaller than the uncertainty in density measurement, it can provide one plausible reason to the deviation of modeling results from experimental data. With this being noted, from thermal fluids considerations, the relative variation in density along the process is the more influential effect.

The variation of viscosity with temperature is also expected to play a key role affecting surface deformation, where the viscosity of the glass forming material increases by 14 orders of magnitude during cooling from the initial temperature to glass transition. In this study, the viscosity is represented by a single exponent term, while a second exponential term could potentially yield larger modeled displacements at higher temperatures, and more moderate displacements with the decreasing temperature [42]. Of course, the single exponential model of viscosity with temperature is the simplest to implement [43], while the knowledge about actual physical behavior of glass-forming CPAs is only sparsely available. While it is possible to keep tweaking the HCPA properties to match a particular experimental dataset, the general conclusion is that the deformation during vitrification is highly dependent on the variation in thermophysical properties.

### Solid mechanics effects on surface deformation

**Fig 7:**
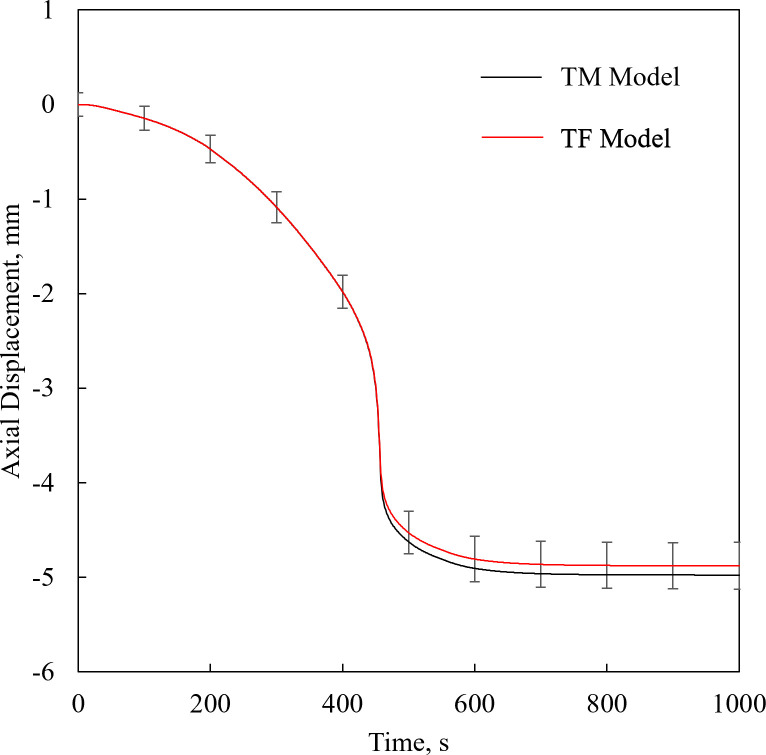
2D TF vs TM model. Comparison of displacement histories along the centerline of the container between the 2D TM and TF 2D models for a cooling rate of 20F0B0C/min. The error bars refer to the uncertainty in experimental measurements for reference, although the displayed results are of FEA.

To compare the simplified TF model with the more comprehensive TM model, Fig 7 displays the displacement histories along the centerline of the cuvette, *u*_*s*_, for a 2D special case having a cooling rate of 20°C/min and minimum temperature of -125°C. The difference in the maximum displacement between the models is less than 1.5%, indicating that the surface deformation is primarily affected by material flow when the viscosity level is relatively low, while material deformation due to mechanical stress may have marginal contribution to the overall deformation.

Recall that the temperature gradients and thermal expansion mismatch across the CPA domain may give rise to mechanical stress [7, 21], while the total strain rate in the TM model is the sum of elastic, thermal and viscous strain rates, Eq (12). In this model, the viscous strain rate in the CPA is significant at high temperatures only, when the viscosity is very low, and it is insignificant at low temperatures, when the viscosity is very high [7]. Furthermore, the thermal expansion in a free of constraints solid due to thermal expansion [44] is much smaller than in a liquid [45] under similar temperature changes. Hence, the deformation in the CPA due to fluid flow is significantly higher than the deformation when the CPA behaves like a solid. It follows that the shape and magnitude of deformation in the vitrifying material can be closely approximated with the TF model, while neglecting solid-mechanics effect. Consistently, stress at high enough magnitudes to affect the structure of the material might occur only at very low temperatures and can be approximated while neglecting earlier material flow.

While the difference in $u_{\underset{=}{s}}$ between the TF and TM models is less than 1.5%, the computation time required for the solving the problems is 26 min and 4.5 hours, respectively, using an Intel Core i7-9700 machine (8-core, 12 MB cache, 4.7 GHz). This opens new opportunities for more computationally affordable modeling, when activating the significant parts of the model based on local criteria, while transitioning from the TF model to the TM model as appropriate. This time saving is of paramount importance when modeling complex organ geometries, and larger container volumes to accommodate them.

### Effect of container shape

**Fig 8:**
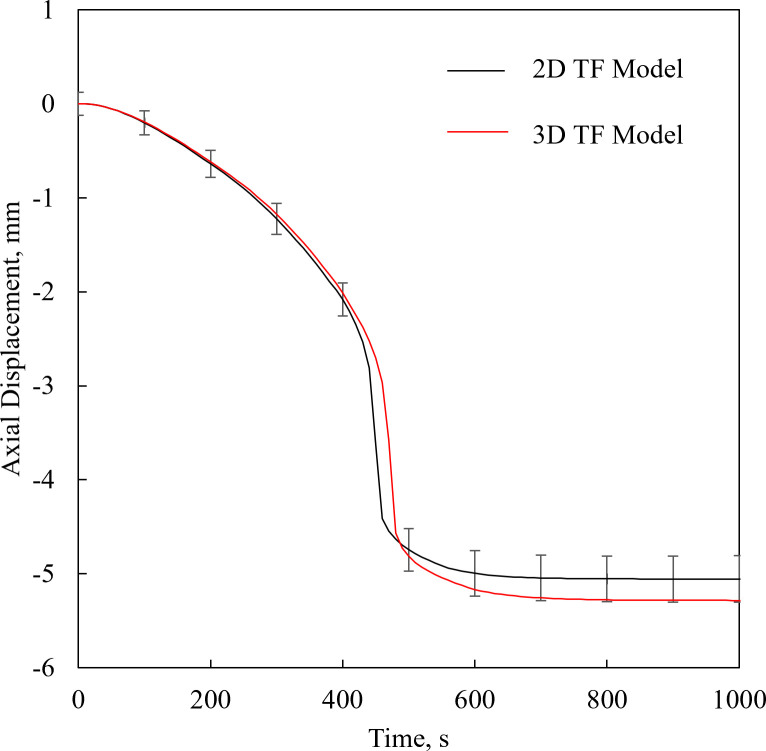
TF 2D vs 3D model. Comparison of displacement histories along the centerline of the container between the TF 2D and 3D models for a cooling rate of 20F0B0C/min. The error bars refer to the uncertainty in experimental measurements for reference, although the displayed results are of FEA.

Fig 8 displays the displacement histories for the 2D and 3D TF models, while maintaining the above thermal protocol. The modeled deformations follow the same trend in both cases, with a maximum difference of 4.1% at final temperature, which is less than the uncertainty in the displacement measurements. The computation time required for the 2D and 3D models is 26 min and 3.5 h, respectively. It follows that simplifying the 3D problem as a 2D case leads to runtime acceleration of an order of magnitude. While this approximation may be good only for a selected set of cases, its effect on runtime is dramatic. Of course, solving the full-blown 3D TM problem requires tremendous computer resources, which may be impractical for complex organ geometries and large-size vitrification.

### Summary and conclusion

A TM mathematical model has been presented recently to investigate thermo-mechanical effects associated with large deformations during cryopreservation by vitrification [11]. The TM model is formulated to solve the coupled problem of heat transfer, fluid mechanics, and solid mechanics. A simplified TF model for the analysis of the same large deformation during vitrification is presented in this study. The main difference in the applications of both models is that the TM model is design for the analysis of thermo-mechanical effects, while the TF model is design to capture the deformation of the vitrified material only. The rationale behind the simplified approach is that large deformations during vitrification occur only in higher cryogenic temperatures, when the low viscosity material can easily flow but cannot sustain high mechanical load. Conversely, significant mechanical stresses develop in low cryogenic temperatures only, due to the exponentially increased viscosity, which results in small deformations. The mathematical solution is obtained by simultaneously solving coupled heat transfer and fluid mechanics problems, using the commercial FEA code COMSOL Multiphysics. The computation results are validated against experimental measurements of axial displacement in the free surface of a CPA-filled cuvette during cryomacroscopy experiments.

For the experimental validation of the mathematical model, six different cases are studied with various cooling rates and final temperatures, representative of cryopreservation applications. The investigated cooling rates are in the range of 10°C/min to -25°C/min, while the final temperature is in the range of -115°C to -135°C. Generally, experimental results demonstrate that the extent of deformation increases with increasing cooling rate, and independently with the decreasing final temperature.

Comparison of the simplified TF model with the more comprehensive TM model proposed previously [11] indicates that the deformation at the CPA surface is primarily due to material flow associated with relatively low viscosity, when the contribution of solid mechanics effects is marginal. Conversely, significant mechanical stress may develop when the material flow is constrained by high viscosity. This suggests that cost-effectiveness in computation of vitrification processes can come about by activating only the significant parts of the TM model based on the localized thermal conditions.

While this study is focused on the surface deformation of CPA contained in a cuvette, the model proposed in this study is applicable to the analysis of deformations in other complex systems, such as the pillow-shaped cryobag. Accounting for the presence of tissue specimens in the CPA domain can be accomplished straightforwardly by further varying the thermophysical properties across the domain, providing specific data on their thermophysical properties. Finally, this study demonstrates the high sensitivity of material deformation not only to the thermophysical property values, but also to the variation of these properties with temperature. This study highlights the unmet need to expand the databases on thermophysical properties of CPA-loaded tissues and organs.

## Supporting information

S1 File (XLSX)
